# Analysis of the Different Lymphatic Drainage Patterns during Sentinel Lymph Node Biopsy for Skin Melanoma

**DOI:** 10.3390/jcm10235544

**Published:** 2021-11-26

**Authors:** Roberto Cirocchi, Giulio Metaj, Michela Cicoletti, Fabrizio Arcangeli, Angelo De Sol, Giulia Poli, Paolo Bruzzone, Sara Gioia, Christos Anagnostou, Fabio Loreti, Simona Francesconi, Linda Ricci, Maria Elena Laurenti, Andrea Capotorti, Marco Artico, Vito D’Andrea, Brandon Michael Henry, Piergiorgio Fedeli, Luigi Carlini

**Affiliations:** 1 Department of Surgery, S. Maria Hospital, University of Perugia, 05100 Terni, Italy; roberto.cirocchi@unipg.it (R.C.); a.desol@libero.it (A.D.S.); luigi.carlini@unipg.it (L.C.); 2 Dermatologic Clinic, S. Maria Hospital, University of Perugia, 05100 Terni, Italy; m.cicoletti@aospterni.it (M.C.); f.arcangeli@aospterni.it (F.A.); 3 Section of Pathology, Department of Medicine and Surgery, University of Perugia, 06132 Perugia, Italy; poligiulia.mail@gmail.com; 4 Department of General and Specialist Surgery “Paride Stefanini”, Sapienza University, 00100 Rome, Italy; paolo.bruzzone@uniroma1.it; 5 Azienda Ospedaliera Santa Maria Terni, Legal Medicine, University of Perugia, 05100 Terni, Italy; s.gioia@aospterni.it; 6 Nuclear Medicine Service, “S. Maria” Hospital, 05100 Terni, Italy; chr.anagnostou@yahoo.it (C.A.); f.loreti@aospterni.it (F.L.); 7 Pathology Unit, Azienda Ospedaliera S. Maria di Terni, University of Perugia, 06121 Perugia, Italy; s.francesconi@aospterni.it (S.F.); l.ricci@aospterni.it (L.R.); me.laurenti@aospterni.it (M.E.L.); 8 Department of Mathematics and Informatics, University of Perugia, 06121 Perugia, Italy; andrea.capotorti@unipg.it; 9 Department of Sensory Organs, “Sapienza” University of Rome, 00100 Rome, Italy; marco.artico@uniroma1.it; 10 Department of Surgical Science, “Sapienza” Università di Roma, 00100 Rome, Italy; vito.dandrea@uniroma1.it; 11 Cardiac Intensive Care Unit, The Heart Institute, Cincinnati Children’s Hospital Medical Center, Cincinnati, OH 45229, USA; Brandon.henry@cchmc.org; 12 School of Law, Legal Medicine, University of Camerino, 62032 Camerino, Italy; piergiorgio.fedeli@unicam.it

**Keywords:** melanoma, sentinel lymph node, biopsy

## Abstract

In the last two decades, studies of lymphoscintigraphy imaging in lymphatic mapping reported an extreme heterogeneity of skin lymphatic drainage of some skin area, in contrast with the previous scientific literature. The aim of this study was to investigate the presence of any correlations between the topographical location of cutaneous melanoma and the topographical location of sentinel lymph nodes. Data from 165 patients undergoing sentinel lymph node biopsy between January 2013 and May 2021 were analyzed, demonstrating that melanomas in the Lumbar region presented a significant more heterogeneous drainage by site than those in the Scapular region (*p* < 0.01) and that melanomas in the Subscapular region were significantly more heterogeneous by laterality (unilateral vs. bilateral) than those in the Scapular region (*p* < 0.05). Results of this study supported the evidence of multiple lymphatic drainage as regards the sentinel node biopsy performed in skin melanoma located on the dorsal subscapular region and lumbar region. For this reason, the association of preoperative lymphoscintigraphy with another imaging evaluation is needed in these critical cutaneous areas. Recent technical developments enabling fluorescence lymphography together with indocyanine green have significantly improved the visualization of lymphatic drainage patterns at a microscopic level. In the preoperative phase, any doubt can be resolved by associating the SPET-CT scan to lymphoscintigraphy, while during the intraoperative phase, an additional evaluation with indocyanine green can be performed in doubtful cases. The aim of the duplex lymphatic mapping (pre and/or intraoperative) is an accurate search of sentinel nodes, in order to reduce the rate of false negatives.

## 1. Introduction

Few data are reported in the literature about the lymphatic vessels draining the skin. Historically, the research of Sappey represents a milestone in this topic [1]: in 1874, he evaluated the lymphatic drainage through injecting mercury in the interstitial tissues and lymphatic vessels during cadaveric dissection.

Anatomical studies of the lymphatic system with the mercury injection technique were widely performed from the 17th to 20th centuries, but this practice was later discontinued for more than 100 years due to mercury’s toxicity. For over a century, the thesis of Sappey concerning the lymphatic vessels draining the skin was generally accepted [1]. Later, this hypothesis was revolutionized by the use of lymphoscintigraphy (LS) imaging in lymphatic mapping studies performed during sentinel lymph node biopsy of patients with melanoma [2]. In the last two decades, recent studies of LS imaging in lymphatic mapping described an extreme heterogeneity of skin lymphatic drainage [3,4] in contrast with Sappey’s rules; these different results are very significant for the melanoma of the torso, in which Sappey, and successively Sugarbaker [5], reported that the lymphatic drainage of the trunk drains to the homolateral side (right or left) and that the location of higher (axilla) or lower (groin) lymphatic station is based on a transverse plane from the umbilicus anteriorly to the level of the L2 vertebra posteriorly. Modern data obtained using LS may modify the conventional lymphatic dorsal drainage pathway [6]. Successively, the use of SPECT/CT imaging has improved the sensibility to identify sentinel lymph nodes (SLNs) than those found by planar lymphoscintigraphy; “especially in the trunk and in the head and neck region, where a non-negligible rate of false-negative SLNB results have been reported” [7].

Suami et al. [8] have recently developed a new imaging method of the lymphatic system in cadaveric specimens by hydrogen peroxide, radiocontrast medium and microinjection techniques. These authors have been able to demonstrate that superficial lymphatic vessels run straight toward the corresponding lymph nodes with fewer interconnections than blood vessels and without connections between superficial and deep lymph-collecting vessels. Furthermore, the superficial lymph collecting vessels do not overlap and divide the skin into territories that correlate with their lymph basins. Suami et al. found another type of lymph vessel running in the subcutaneous tissue but perforating the topographical location of cutaneous melanoma and the location of sentinel lymph nodes.

The aim of this study was to investigate the presence of any correlations between the topographical location of cutaneous melanoma and the topographical location of sentinel lymph nodes.

## 2. Materials and Methods

### 2.1. Inclusion and Exclusion Criteria

#### 2.1.1. Patients Included

Male and female patients over 18 years of age who underwent cutaneous exeresis for Melanoma and subsequent Sentinel Lymph Node Biopsy (SLNB) at the Unit of General Surgery at the “S. Maria” Hospital in Terni (Italy) between January 2013 and May 2021.

#### 2.1.2. Exclusion Criteria

Patients who had cutaneous exeresis for Melanoma with stage pT1b or higher but without available SLNB, or patients treated for Melanoma with stage lower than pT1b according with AJCC8th (melanoma with a thickness between 0.8 mm to 2 mm with no ulceration or melanoma with a thickness less than 0.8 mm with ulceration).

### 2.2. Outcome

The analysis of the correlation between the topographical location of cutaneous melanoma and the intraoperative identification of sentinel lymph nodes.

### 2.3. Interventions

The SLNB technique initially used intradermal injections of vital blue dye and was later modified with the addition of radiolabeled colloid injections at the site of the primary cutaneous melanoma. Lymphoscintigraphy was performed with intradermal injection of 55 MBq of Technetium-99 m-nanocolloid (Nanocoll, GE Healthcare, Gipharma, Saluggia, VR, Italy; 2019–2021: Nanotop, Rotop Parmaka Gmbh, Dresden, Germany) the same day of the surgical procedure. The colloid was injected in a total volume of 0.4 mL in four intradermal deposits of 0.1 mL each, which were located on both sides of the surgical scar. Dynamic images of the corresponding anatomical region and their adjacent lymphatic basins were acquired at 30 s per frame for 5 min with a total of 10 frames. Afterwards, anterior, lateral, and oblique projections were acquired for 5 min each, using a dual-detector gamma camera. The studies were integrated with a SPECT-CT acquisition (GE Infinia Hawkeye SPECT-CT). Images of each patient were reviewed by the operating surgeon and the nuclear medicine specialist.

### 2.4. Satisical Analysis

Standard descriptive analyses (frequencies, medians, and standard deviations) have been performed on data of the different groups.

To test effective heterogeneity for site or laterality of lymph nodes on each topographical location, two statistically similar analyses (one for site and another for laterality) were performed by choosing the Shannon entropy index as test statistic, while the Hutcheson *t*-test was performed to check the significance of the pairwise differences among groups. Tests have been performed through the open-source R statistical software (version 4.0.2, The R Fundation for Statistical Computing c/o Institute for Statistics and Mathematics, Wirtschaftsuniversitat Wien, Vienna, Austria).

Only meaningful comparisons between groups that had expressed some heterogeneity are reported in the Results section.

## 3. Results

**Figure 1:**
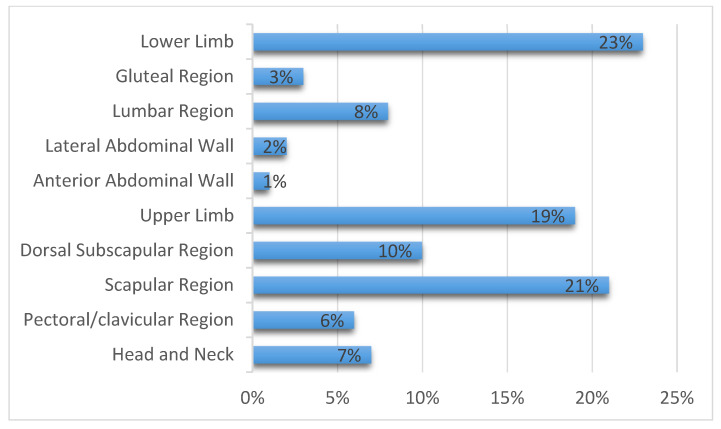
Localization of primary melanoma included in this study and their frequency.

This retrospective observational study collected data on 165 consecutive patients with cutaneous melanoma (Figure 1).

All patients included in this study, underwent SLNB for cutaneous melanoma with a stage equal or greater than pT1b according to AJCC 8th (melanoma with a thickness between 0.8 mm to 2 mm without ulceration or melanoma with a thickness less than 0.8 mm with ulceration); cases previously performed with the last AJCC 7th update were re-staged with the new classification.

The characteristics of the enrolled patients were as follows:

- sex: 77.28% male (111) and 32.72% female (54);
- median age and standard deviation: 62 ± 15.15

1. The Breslow depth was reported in 157 cases (95.15%), the higher number of patients had a stage II of Breslow (48.4%) and a stage III (27.4%) (Table 1).
2. The T classification of melanoma, based on the thickness of the primary tumor and presence or absence of ulceration, is reported in 156 patients (94.23%), the higher number of patients have a stage T1b (32.7%) and T2a (29.5%) (Table 2).

**Table 1:** Breslow depth.

Stage	Depth	Number of Patients (%)
Stage I	0.75 mm or less	18 (11.4%)
Stage II	0.76 mm–1.50 mm	76 (48.4%)
Stage III	1.51 mm–4.00 mm	43 (27.4%)
Stage IV	>4 mm	20 (12.8%)

**Table 2:** T classification of reported Melanomas.

Stage	Depth of Melanoma	Number of Patients (%)
T1b	≤1.0 mm in thickness with ulceration or mitoses ≥1/mm^2^	51 (32.7%)
T2a	1.01–2.0 mm in thickness without ulceration	46 (29.5%)
T2b	1.01–2.0 mm in thickness with ulceration	11 (7.0%)
T3a	2.01–4.0 mm in thickness with ulceration	14 (9.0%)
T3b	2.01–4.0 mm in thickness without ulceration	18 (11.5%)
T4a	>4.0 mm in thickness without ulceration	5 (3.2%)
T4b	>4.0 mm in thickness with ulceration	11 (7.0%)

### 3.1. Data and Analysis

**Figure 2:**
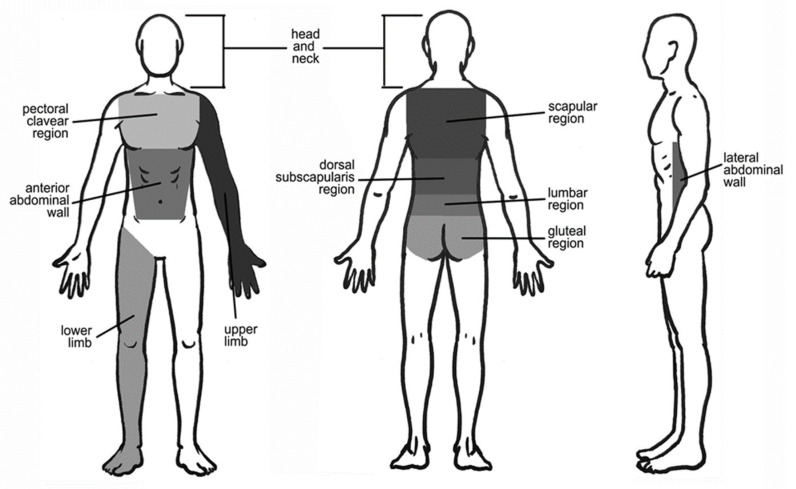
Anatomical boundaries of the topographic location regions of the melanomas studied.

The topographical site of localization of primary melanomas has been studied individually and correlated to the topographical region of the SLNB. These cutaneous melanomas presented a peculiar lymphatic drainage based on the different topographic locations (Figure 2); for this reason, two broad categories were identified: homogeneous drainage group and heterogeneous drainage group. To figure out which group to put each region into, we followed three key points:

- regions draining at least 90% of the time into the same group of lymph nodes were considered homogeneous;
- regions of the lateral wall of the abdomen and gluteus, considering the low number of cases, and not presenting a significantly heterogeneous pattern were classified as homogeneous;
- regions that were shown to have multiple and/or bilateral drainage in significant numbers were identified as heterogeneous.

#### 3.1.1. Homogeneous Drainage Group

**Figure 3:**
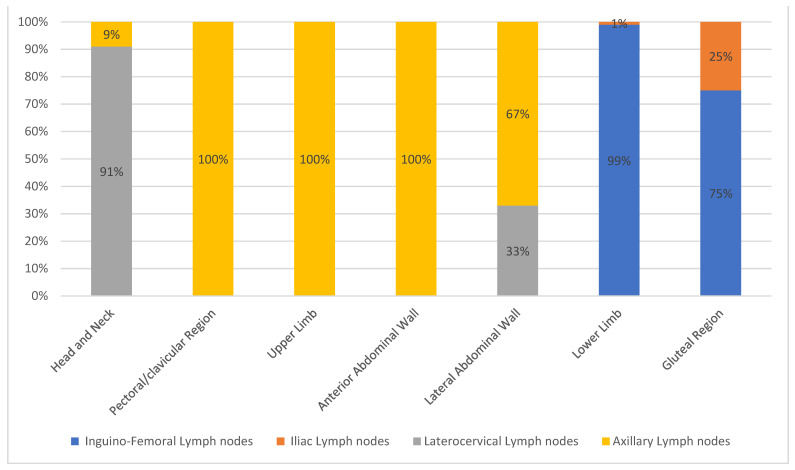
Lymphatic drainage of “Homogeneous drainage group” melanomas.

1. *Head and neck*. Twelve cutaneous melanomas were located in this region (7.27% of all included patients) (Figure 1). The greatest number of cutaneous melanomas drained into the laterocervical lymph nodes (91.17%) and only one patient drained into the axilla (8.3%) (Figure 3). The pT evaluation reported two pT1b, three pT2a, three pT3b, one pT4a, and one pT4b case(s); in two cases the pT evaluation was not reported (Table 3).
2. *Pectoral/clavicular region*. In this area, 11 cutaneous melanomas were located (6.66% of all included patients) (Figure 1) and, in all cases, the lymphatic drainage was at the level of the homolateral axillary lymph nodes (100%) (Figure 3). In this group, two pT1b, three pT2a, one pT2b, one pT3a, one pT3b, and two pT4b cases were reported; in one case pT evaluation was not reported (Table 3).
3. *Upper limb*. In this region, 32 cutaneous melanomas were localized (19.39% of all included patients) (Figure 1) and in all cases the lymphatic drainage was at the level of the homolateral axillary lymph nodes (100%) (Figure 3). At this level, the location was as reported in Table 3.
  - *Arm*. In twenty-seven patients, the cases were five pT1b, eight pT2a, seven pT2b, two pT3a, two pT3b, and two pT4b. In one case, pT evaluation was not reported.
  - *Forearm*. Five patients were reported in this group: one case of pT1b, pT2b, pT3a, and pT3b, respectively. In one case, pT assessment was not reported.
4. *Anterior abdominal wall*. In this region, one cutaneous melanoma (Figure 1) with a pT1b stage was located, draining to the homolateral axillary lymph nodes (Figure 3) (Table 3).
5. *Lateral abdominal wall*. Three cutaneous melanomas were located on this region (Figure 1), draining to homolateral axillary lymph nodes in two cases (66.66%) and to homolateral laterocervical lymph nodes (33.33%) in one case (Figure 3). In this group, two pT2a and one pT3b were reported (Table 3).
6. *Lower limb*. Here, 38 cutaneous melanomas were localized (23.03% of all included patients) (Figure 1) and in all cases the lymphatic drainage was at the level of the homolateral iliac–femoral lymph nodes (100%) (Figure 3). By means of a functional, not simply anatomical, assessment, it is possible to consider these two lymph node stations, iliac and femoral, as a single drainage group: the iliac–femoral lymph nodes.
  At this level, the location was as seen in Table 3.
  - *Thigh*. In this region there were 16 patients with SLNB at the homolateral inguinal–femoral lymph node. The pT assessment was pT1b in six cases and pT2a in five cases, with one case of pT3a, two cases of pT3b and two cases of pT4b.
  - *Knee*. One patient with SLNB at the homolateral inguinal–femoral lymph node. This patient had a pT1b stage.
  - *Calf*. Sixteen patients with SLNB to the homolateral inguinal–femoral lymph node and also at the iliac lymph node in three cases (18.75%). In these patients with SBLN at the iliac lymph node the pT evaluation was pT1b in two cases and pT3a in one case; in patients with only SBLN at the homolateral inguinal–femoral lymph node, there were two cases of pT1b, seven cases of pT2a, and two cases of pT3a and pT4b.
  - *Foot*. Five patients with SLNB at the homolateral inguinal–femoral lymph node. In these patients, the pT assessment was a pT1b in three cases and a pT3a in one case; in one case no pT assessment was reported.
7. Gluteal region. In this area, four cutaneous melanomas were located (2.42% of all included patients) (Figure 1) and in three cases the lymphatic drainage was to the homolateral inguinal–femoral lymph nodes (75%); only in one patient was drainage to the iliac lymph node reported (25%) (Figure 2). In this group, three pT1b were reported, and in one case pT evaluation was not reported (Table 3). According to the above functional evaluation, it can be stated that the drainage of cutaneous melanomas of the gluteal region was in 100% of the cases at the iliac–femoral lymph nodes.

#### 3.1.2. Heterogeneous Drainage Group

**Figure 4:**
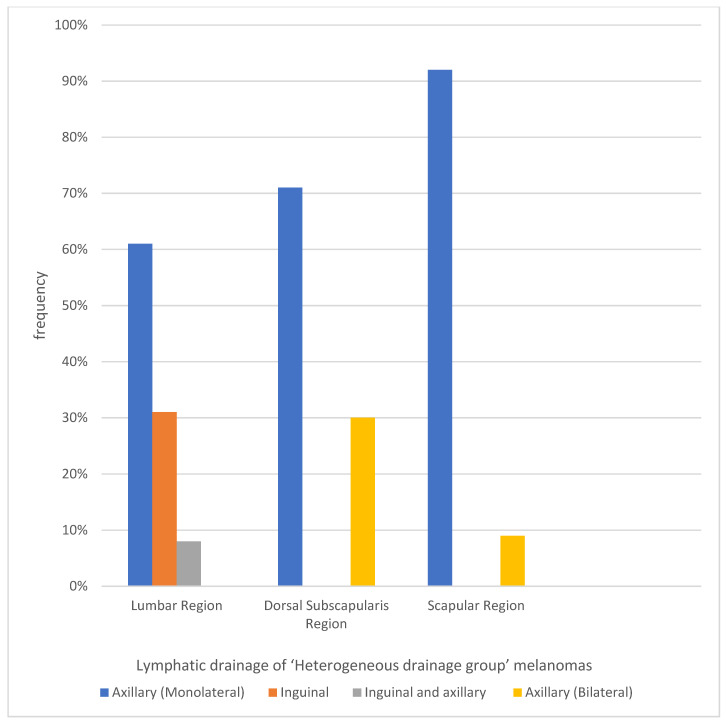
Lymphatic drainage of “Heterogeneous drainage group” melanomas.

1. *Lumbar region*. In this area, 13 cutaneous melanomas were located (7.88% of all included patients) (Figure 1). Lymphatic drainage was very heterogeneous: eight patients to axillary lymph nodes only (61.53%), one patient to axillary and inguinal lymph nodes (7.69%), and four patients to inguinal level only (30.77%) (Figure 4). In this group, five pT1b, two pT2a, two pT3a, and two pT4a cases were reported; pT evaluation was not reported in two cases (Table 3).
2. *Dorsal subscapularis region*. Seventeen cutaneous melanomas were localized to this region (10.3% of all included patients) (Figure 1). The skin melanoma drained into the homolateral (70.59%) or bilateral (29.41%) axillary lymph nodes (Figure 4). The pT evaluation reported three pT1b, four pT2a, two pT2b, two pT3a, three pT3b, and two pT4a cases; in one case the pT evaluation was not reported (Table 3).
3. *Scapular region*. Thirty-five cutaneous melanomas were located in this region (21.21% of all included patients) (Figure 1). Drainage of the skin melanoma was to the homolateral axillary region in 91.43% of the cases; bilateral drainage was reported in only three cases (8.57%) (Figure 4). The pT evaluation reported eight pT1b, nine pT2a, one pT2b, three pT3a, four pT3b, two pT4a, and six pT4b cases. In one case the pT evaluation was not reported (Table 3).

**Figure 5:**
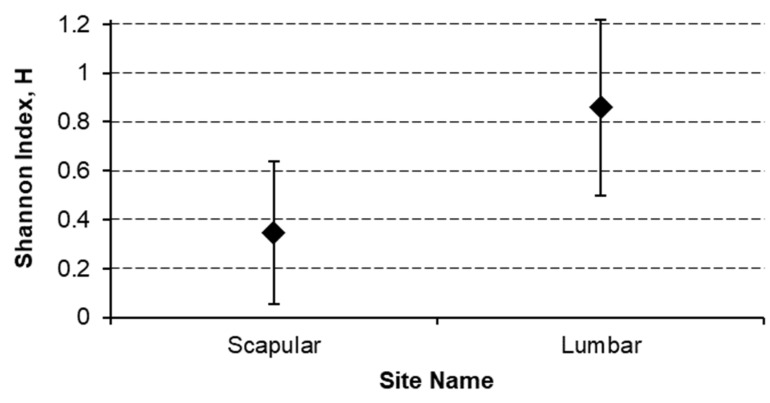
Lymphatic site heterogeneity comparison.

**Figure 6:**
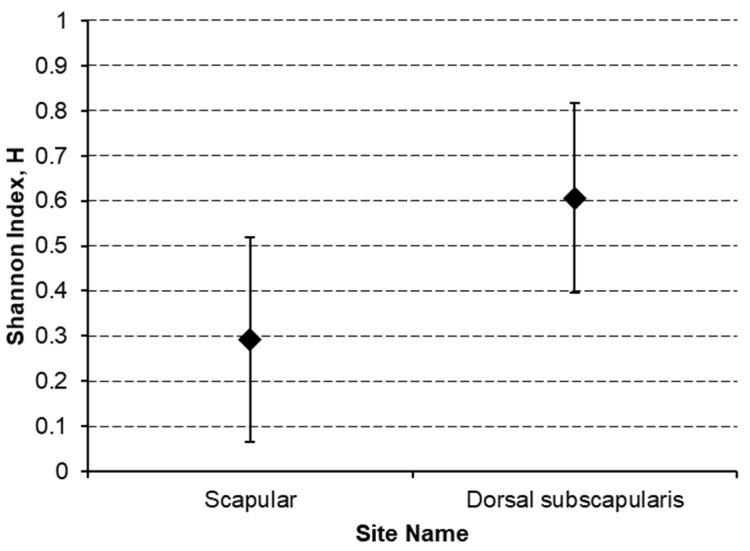
Lymphatic laterality heterogeneity comparison.

To measure heterogeneity, the Shannon entropy index H has been computed for either the site or the laterality on these last three groups and differences between H values has been tested through the Hutcheson *t*-test, by obtaining the following results:

- Lumbar region has a most significant (*p* < 0.01) heterogeneous site of lymph node region drainage with a Shannon index of 0.876 (CI 0.500–1.217) with respect to the 0.347 (CI 0.055–0.639) of the Scapular region (Figure 5).
- Dorsal subscapularis region presented the most significantly (*p* < 0.05) heterogeneous lymphatic laterality drainage (homolateral vs. bilateral) with a Shannon index of 0.606 (CI 0.395–0.816) against the 0.293 (CI 0.065–0.520) of the Scapular region (Figure 6).

## 4. Discussion

In this observational study, we carried out an analysis of the different lymphatic drainage pathways of skin melanomas.

Lymphatic drainage was homogeneous for the lower limbs, as well as for the gluteal region which, similar to the lower limb, constantly drained to the iliac–femoral level. In addition, the popliteal lymph node station did not have any clinical value in this series of patients; in fact, none of the 21 patients with cutaneous melanoma located below the knee demonstrated lymphatic drainage to the popliteal station. The lymphatic drainage of the upper limb followed the same pattern of extreme homogeneity, involving the homolateral axillary level in all melanomas of this site. Drainage of the anterior wall of the thorax (including pectoral and clavicular region) was homogeneous and always occurred at the axillary level, while drainage of the back was heterogeneous in terms of location and laterality. In contrast, there was a high heterogeneity in the regions of posterior torso (scapular, subscapular, and lumbar). In the scapular and dorsal subscapular region, cutaneous melanomas drained to homolateral axillary region in 88.57% and 70.59% of cases, and to bilateral lymph nodes in 8.57% and 29.41% of cases, respectively. In the lumbar region, there was a considerable heterogeneity as regards the site of the lymphatic drainage: in fact, in 61.53% of cases there was drainage to the axillary lymph nodes, and in 30.77% there was drainage at the inguinal level; in 7.69% of cases, drainage occurred in both sites.

This asymmetric drain had already been reported from Leong [9] and summarized from Marone [10] as follows: “If a melanoma is located within 2.5 cm of the midline, the drainage can occur to either side or both sides and is not considered discordant. Similarly, for melanomas located within 2.5 cm of the Sappey line, drainage can occur to the homolateral groin, axilla, or both”. The data collected in this study allow us to suggest particular attention during preoperative lymphoscintigraphy in case of cutaneous melanomas located in the subscapular region, where it will be necessary to carefully check the uptake; in addition, particular attention should also be paid to the evaluation of the lymphatic drainage of the lumbar lymph nodes in which the axillary and inguinal region must always be explored. In the cases with heterogeneous lymphatic drainage (lumbar region, dorsal scapular and subscapularis) it would be advisable to associate the preoperative lymphoscintigraphy with an intraoperative indocyanine green fluorescence (ICG) assessment in order to reduce the risk of false negatives for the aforementioned reasons [11]. In fact, the recent technical developments enabling fluorescence lymphography together with indocyanine green have significantly improved the visualization of lymphatic drainage patterns at a microscopic level [12].

Similar results were found in a cooperative study performed in New Zealand and Australia on a database of 5232 patients [13]. Reynolds et al. reported the results analyzed through an online software tool which allows interactive displays showing draining node fields from regions of skin, which should be used to compare heterogeneous drainage patterns with this study’s findings. In effect, the melanoma of skin near the midline and waist of the posterior torso has a very heterogeneous lymphatic drainage patterns (left axilla 71.4%, right axilla 52.4%, intercostal 4.8%, paravertebral 4.8%, retroperitoneal 4.8%, left groin 66.7%, right groin 23.8% and Interval Node 9.5%). Conversely, the melanoma of skin near the left axilla drains in 100% of axillary lymph nodes.

Our analysis showed the same results for the skin of the shoulder region that drains symmetrically and for the skin of the subscapular region and lumbar regions that have a heterogeneous drainage. In our study, a higher rate of bilateral drainage of subscapular region emerged (70.59% homolateral and 29.41% bilateral in axillary region), along with asymmetrical drainage (groin and axilla) of the lumbar region (61.53% in axillary lymph nodes, 30.77% in inguinal lymph nodes, and 7.69% in axillary and inguinal lymph nodes).

**Figure 7:**
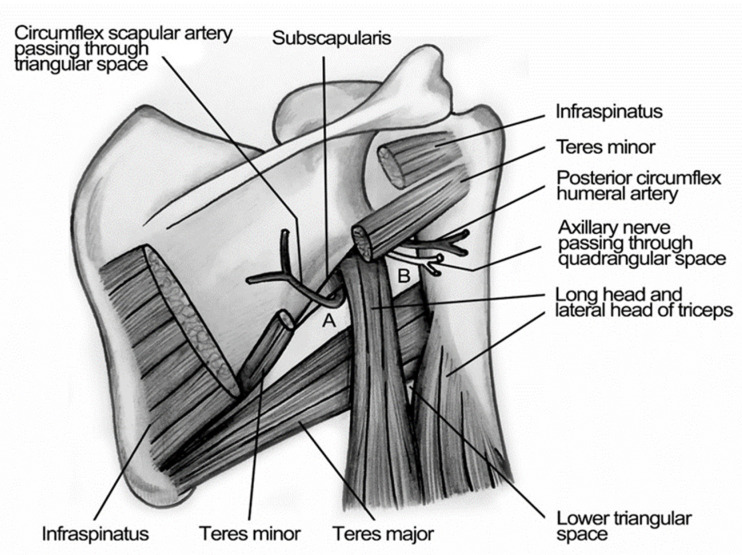
Illustration of Triangular Intramuscular Space (TIS). Subscapular spaces. A: Triangular Intermuscular Space (TIS); superior border: lower margin of Teres Minor, inferior border: upper margin of Teres Major, lateral border: medial margin of the long head of Tricepts. B: Quadrangular Space of Velpeaum; superior border: inferior margin of Teres Minor, inferior border: superior margin of Teres Major, medial border: medial margin of the long head of Triceps, lateral border: medial margin of shaft of Humerus.

The small sample size is the principal limitation of our study: this bias is associated with the absence of uncommon site of lymphatic drainage from shoulder and upper trunk as the neck (6.2%) [14,15]. This bilaterality of lymphatic drainage of subscapular region is very difficult to explain according to our classical anatomical clinical knowledge. A hypothesis to explain this drainage is a lymphatic route through the triangular intermuscular space (TIS), through which passes the descending circumflex scapular artery, vein, and lymphatics (Figure 7). This triangular space, located lateral to the scapula, is bounded by the teres major inferiorly, the infraspinatus, teres minor, and subscapularis superiorly, and the long head of the triceps laterally [16].

This intermuscular space was first described by Uren [17] in 1996 as the gateway to the axilla, associated with an important site of recurrent melanoma [18]. Successively, Hennessey [16] improved the description of TIS and categorized three different lymphatic drain pathways of scapular and subscapular regions:

- Pattern 1: it separates lymphatic channels draining to the axilla directly, bypassing the TIS node.
- Pattern 2: lymphatic channels draining first to the TIS node, then to a homolateral axillary node.
- Pattern 3: lymphatic channels draining to the TIS and the axilla, as well as separate channels that drain to the axillary nodes and bypassed the TIS nodes.

Furthermore, the bilaterality can be explained with the presence of lymphatic network located between posterior muscles of the trunk and subscapular arteries that can present anatomical variations [19,20,21]. It was not possible to explain the heterogeneity of lymphatic drainage of torso; this condition was previously described by Bonmarchand [22], but the physiopathological mechanism is still unknown. Conversely, classic anatomical literature reported robust evidence that lymph from the skin lumbar region drains into the subscapular lymph nodes [23]. Otherwise, in groups with homogeneous lymphatic drainage (drainage of the anterior and lateral abdominal wall, upper and lower limb, pectoral and clavicular region, and head–neck region), the two above detection methods could be combined to reduce the number of false negatives in case of larger lymph nodes. The use of the dye, according to our knowledge, allows us to highlight the lymphatic pathway that leads from melanoma to the SLN. Considering the case of large metastases in the SLN that can inhibit tracer accumulation in these nodes, the use of the radioactive tracer alone would generate an error of assessment, as once it reaches at the lymph nodes involved with tumor cells it will not be able to enter it, and would therefore deposit in non-sentinel non-metastatic lymph nodes, thus creating a false negative [24].

As suggested from EANM practice guidelines a “preoperative ultrasonography should be performed as a staging procedure of the nodal basin most likely to be the drainage site of the primary melanoma” [24]. Perhaps, new studies about connection among subgroups of regional lymph nodes can explain this issue. Suami et al. recently studied the lymphatic system by using the hydrogen peroxide, radiocontrast medium and microinjection technique, so they have theorized the “lymphosome” as a new anatomical concept [8,25,26]. Many of our results agree with the “lymphosomes” of the body described by Suami; there is only a divergence about the torso between the heterogeneity of our findings and the well-demarcated lymphatic territory according to the corresponding lymphatic basins reported by Suami.

In common clinical practice, this heterogeneity of lymphatic drainage of the torso is associated with evidence that patients with truncal melanoma with multiple lymphatic drainage are associated with a worse prognosis rather than patients with one homogeneous drainage [27,28].

## 5. Conclusions

Our results can support previous data about heterogeneity of SLN biopsy performed in cutaneous melanoma localized in both dorsal subscapular region and lumbar region. For these reasons, in cases of doubtful sentinel lymph nodes, there is a need to add other imaging evaluations to the preoperative lymphoscintigraphy.

In fact, in the preoperative phase, the combined use of PET-CT and LNS can resolve some uncertainty, whilst an additional evaluation with indocyanine green can be performed during the intraoperative phase.

## Figures and Tables

**Table 3:** pT classification of reported melanomas.

	pT1b	pT2a	pT2	pT3a	pT3b	pT4a	pT4b	Not Reported
Head and Neck	2	3	0	0	3	1	1	2
Pectoral/clavicular Region	2	3	1	1	1	0	2	1
Upper Limb (Arm/Forearm)	5/1	8/0	7/1	2/1	2/1	0/0	2/0	1/1
Anterior Abdominal Wall	1	0	0	0	0	0	0	0
Lateral Abdominal Wall	0	2	0	0	1	0	0	0
Lower Limb (Thigh/Knee/Calf/Foot)	6/1/4/3	5/0/7/0	0/0/0/0	1/0/3/1	2/0/0/0	0/0/0/0	2/0/2/0	0/0/0/1
Gluteal Region	3	0	0	0	0	0	0	1
Lumbar Region	5	2	0	2	0	2	0	2
Dorsal Subscapularis Region	3	4	2	2	3	2	0	1
Scapular Region	8	9	1	3	4	2	6	1

## Data Availability

The data presented in this study are available on request.
